# Integrating Molecular Dynamics Simulations and Single-molecule FRET Spectroscopy: From Computational FRET Estimation to Experimental Data Interpretation

**DOI:** 10.1021/acs.jpcb.5c05660

**Published:** 2026-01-05

**Authors:** Stephanie Sauve, Ehsaneh Khodadadi, Ahmed Shubbar, Ehsan Khodadadi, Mahmoud Moradi

**Affiliations:** Department of Chemistry and Biochemistry, 3341 University of Arkansas, Fayetteville 72701, Arkansas, United States

## Abstract

Molecular dynamics (MD) simulations can characterize biomolecular processes at an exceptional spatiotemporal resolution not able to be accessed experimentally. As the limitations associated with MD simulations lessen and the method advances toward greater capabilities, the simulations are being applied to a wide array of new applications. For example, the integration of MD simulations and single-molecule Förster resonance energy transfer (smFRET) spectroscopy is a newly developing and growing application combining experimental and computational approaches. The integration of these techniques provides valuable insight into the conformational dynamics of biomolecules on an atomic-level, thereby enhancing the understanding of complex biological processes. This review compiles information on simulating FRET dyes and estimating FRET efficiencies from MD simulations and using MD simulations to gain insight into experimental data to shine light on the recent advancements in joining computational and experimental techniques. We discuss notable studies that incorporate the use of both MD simulations and smFRET as well as discuss the challenges that have been faced regarding their integration. The joining of these approaches have provided valuable insights into conformational sampling, binding mechanisms, structural dynamics, and allosteric effects thus far and will continue to advance the understanding of biomolecular dynamics in the future.

## Graphical abstract

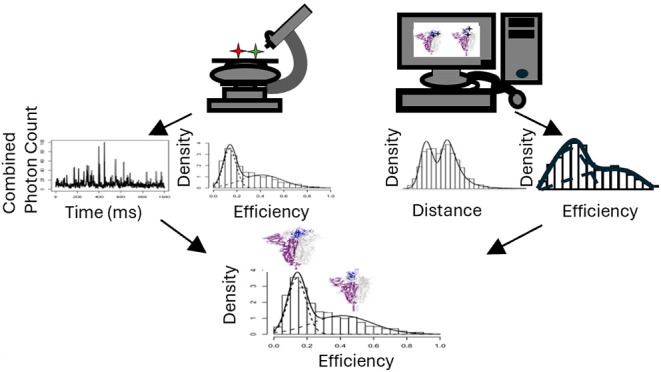

## I Introduction

Molecular dynamics (MD) simulations, often guided and/or validated by experimental techniques, have proven to be valuable in advancing our understanding of the structural and chemo-mechanical properties of various biomolecules.^1–5^ These simulations have a broad range of applications and are able to capture many important biomolecular processes such as ligand binding, conformational state changes, protein folding, and more.^6–14^ Furthermore, MD simulations can be used to refine experimentally obtained structures and have contributed to the understanding that proteins are conformationally dynamic as opposed to being static structures.^15–20^ Trajectories obtained from these simulations can be analyzed to provide specific information on interest, such as free energy changes associated with mutations.^15,21–25^

By integrating over short time steps in the femtosecond range, MD simulations typically allow for time scales from the femto- to microsecond level to be studied.^17,26–28^ Such small time scales are often inaccessible during experimental observation; thus, MD simulations provide atomic level detail at unique, pinpoint time scales. Although MD simulations provide immense atomic level details, the simulations have their limitations. First, MD simulations require a starting structure, meaning they cannot be initiated if a structure is not available for the system of interest. Additionally, the force fields used in MD simulations are poorly suited for systems where quantum effects are significant and therefore need refinement when using uncommon chemical species.^29–32^ Adding parameters to force fields is possible, but is often complex and time-consuming.^30–34^ Furthermore, the sampling of the system during the simulation may be limited due to high computational demands.^29,30,35,36^ Despite their limitations, MD simulations are increasingly used and will continue to grow in popularity as these problems are addressed and its applications expand. For example, integrating MD simulations with smFRET methodology is a newly developing and growing approach for data analysis and experimental design.

FRET is a technique that measures the nonradiative energy transfer from fluorescent donor dye molecules to fluorescent acceptor dye molecules via resonant dipole–dipole interactions to ultimately determine the distance between the location of the two dyes.^37–49^ The technique is dependent on the relative orientation of the donor and acceptor dipoles, the absorbance/emission spectra of the dyes, and varies by the interdye distance. For FRET to occur, the emission spectra of the donor dye must overlap with the absorption spectra of the acceptor dye (Figure 1).^47,49–51^

**Figure 1:**
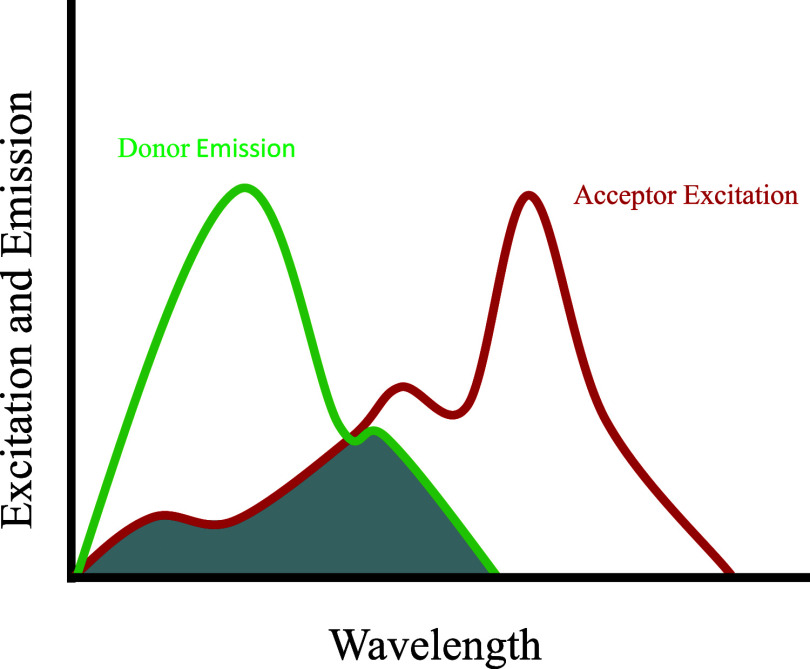
A simplified schematic representation showing the overlap (gray) of the donor fluorophore emission spectra shown in green and the acceptor fluorophore absorption spectra shown in red. The overlap of the spectra must be present for FRET to occur. In practice the actual overlap increases with wavelength.

Energy transfer between the dyes can be measured using FRET because the transfer results in a decrease in the fluorescence of the donor fluorophore and a corresponding increase in the fluorescence of the acceptor fluorophore.^42,46,52^ If energy transfer between the dyes occurs, then the acceptor emits a photon,^45^ with a higher number of acceptor photons emitted signifying a more efficient energy transfer between the dyes. The FRET efficiency (*E*), among other methods, can be estimated based on the ratio of acceptor intensity (*I*_A_) to total emission intensity, which is the sum of the acceptor intensity and the donor intensity (*I*_D_):^43^

1 $$E=\frac{I_{A}}{I_{A}+I_{D}}$$

This equation assumes that the donor and acceptor dyes have equal quantum yields and detection efficiencies, or that the fluorescence intensities have been normalized accordingly. The FRET efficiency is dependent on the distance between the two dyes (*r*). Within certain approximations, the relationship between the *E* and *r* is often simplified according to

2 $$E=\frac{1}{1+{\left(\frac{r}{R_{0}}\right)}^{6}}$$

where *R*_0_ is the Förster radius, which is the distance at which half of the energy is transferred from the donor to the acceptor $\left(E=\frac{1}{2}\right)$ .^37,41,43,45,51,53^ The *R*_0_ is a function of the properties of the dyes and their relative orientation.^37^ It can be expressed by the equation:

3 $$R_{0}\approx 0.211{\left[\kappa^{2}n^{-4}Q_{D}J\left(\lambda \right)\right]}^{1/6}\left(\mathrm{in}\;Å\right)$$

where *n* is the refractive index of the medium (approximately 1.4 for biomolecules in water^54^), *Q*_D_ is the quantum yield of the donor in the absence of the acceptor, κ^2^ is the orientation factor ( $\kappa^{2}=\frac{2}{3}$ assuming the donor and acceptor fluorophores undergo fast, isotropic rotation^55,56^ ), *J*(λ) is the spectral overlap integral between the donor emission and acceptor absorption spectra^53^ (Figure 1) and the constant 0.211 results from

4 $$0.211\approx {\left(\frac{9\;\ln\left(10\right)}{128\pi^{5}N_{A}}\times 10^{28}\right)}^{1/6}$$

where *N*_A_ is Avogadro’s number. In eq 3 κ^2^ describes the relative orientation of the donor and acceptor transition dipole moments. Additionally, *J* has the units of M^–1^ cm^–1^ nm^4^. Under this convention, eq 3 yields (*R*_0_) in Å, and eq 4 is unit-free, as the 10^28^ factor incorporates unit conversions.

To date, FRET technique has been successfully used in biochemistry, polymer science, and structural biology to measure distances in the 1–10 nm range,^37,41,52,57–64^ permitting the use of FRET as a “spectroscopic ruler” (Figure 2).^41–43,46,53,65,66^

**Figure 2:**
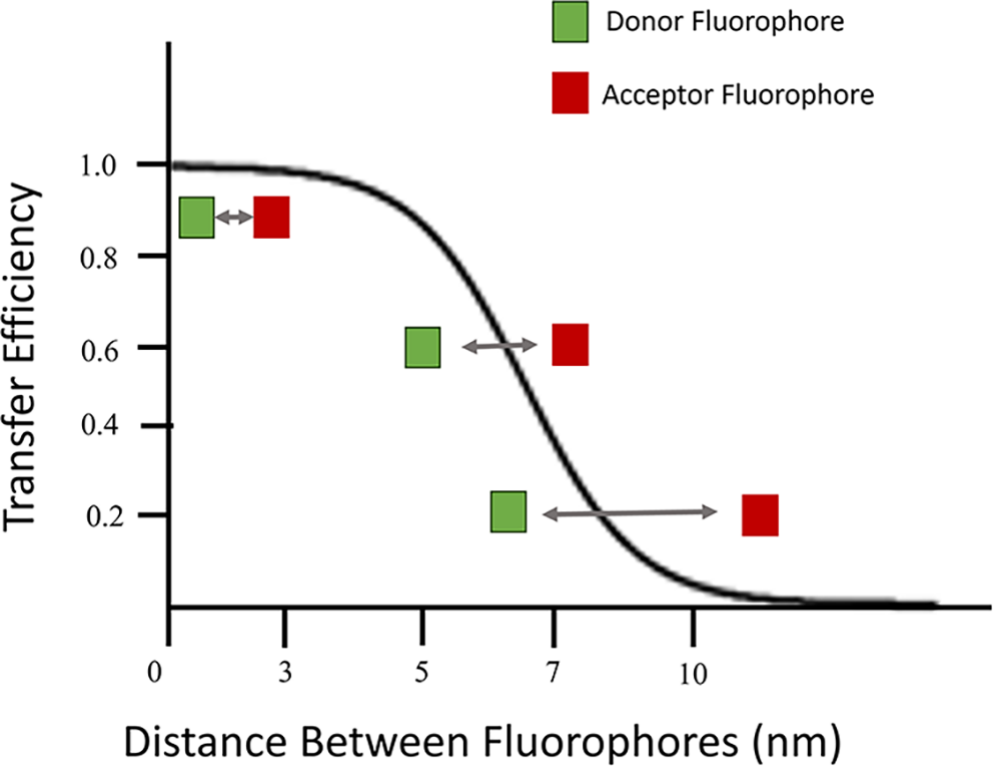
Schematic representation of FRET showing an energy transfer between a donor fluorophore shown in green and an acceptor fluorophore shown in red. The relationship between the distance of the dyes and the efficiency of transfer is shown with the optimal transfer distance being between 1 and 10 nm. The further the dyes move away from each other, the lower the transfer of energy from the donor fluorophore to the acceptor fluorophore.

FRET experiments can be carried out at either the ensemble or single-molecule level. The single-molecule FRET (smFRET) technique was first used in 1996.^37^ smFRET is capable of collecting data for a single pair of donor and acceptor fluorophores that are labeled at site-specific locations as opposed to ensemble FRET which provides data for an ensemble of molecules by concurrently exciting many donor molecules.^45,51,53,67^ In contrast to ensemble FRET, smFRET circumvents population averaging by selectively exciting donor fluorophores and detecting donor and acceptor emissions from individual molecules.^37^ This approach enables the identification of multiple conformational states that are typically masked in bulk measurements. Therefore, utilizing the smFRET technique allows for intermolecular/intramolecular distances to be calculated for studying time-dependent events such as conformational changes of a single molecule.^37,41,43,51,53,68–71^

There have been various studies since mid 2000s that combine the smFRET technique with MD simulations.^72,73^ In these studies, MD simulations are often performed alongside experimental techniques to provide molecular level insight.^74–79^ However, integrating smFRET and MD has been challenging for various technical reasons. Various research laboratories have attempted to address issues regarding how the dyes are simulated^56,80–84^ and how to accurately estimate FRET efficiency^72,85^ from MD simulations. Recently, more advanced MD simulation techniques such as Markov state models (MSMs) have also been employed to help with the integration of smFRET and MD simulations.^45,86–88^

Here, we compile a focused yet diverse set of studies to illuminate how smFRET–MD integration has been carried out to date, with the goal of informing new strategies for more effective integration of the two techniques. We begin by reviewing select studies that focus on classical MD simulations of dye-labeled proteins and the estimation of FRET efficiencies from such simulations. We then turn to select studies that use classical MD to interpret experimental smFRET data, whether through conventional simulations or in conjunction with advanced sampling techniques such as enhanced sampling and MSMs. Finally, we conclude with a summary and a brief discussion of future directions in the field.

## II Simulating FRET Dyes and Estimating FRET Efficiencies from MD Simulations

Incorporating MD simulations with experimental techniques such as smFRET has proven to be a powerful approach for studying the behavior of proteins and gaining insight into protein structure–function relationships. However, there are many factors that need to be considered for the joining of the techniques to be effective and reliable. For instance, simulating the dyes used for the fluorescent labeling and ensuring that these simulations correctly represent the underlying physics may be challenging.^56,82–85^ One must also be mindful of how the data is evaluated when estimating FRET from MD simulations in order to obtain correct interpretations.^56,72,81^ Here we focus on factors that need to be considered when simulating the dyes used in an smFRET setting and presents research that has shown how to correctly interpret smFRET data from MD simulations.

### II.A Simulating Dyes Attached to Proteins

Several studies have utilized MD simulations to explore various aspects of dye dynamics in solution and protein complexes. Some challenges specific to simulating accurate dye behavior have been addressed in these studies. For example, the commonly assumed isotropic dye orientation distribution, where both fluorophores’ transition dipoles are assumed to independently sample all possible orientations equally, is often difficult to verify experimentally.^56,89–93^ Theoretically, the value of κ^2^ can vary between 0 and 4 depending on the relative orientation of the dyes’ dipoles. In addition, for FRET to occur, κ^2^ cannot be 0 as that would imply that the dipoles are perpendicular to each other.^53,56^ The commonly assumed value of $\kappa^{2}=\frac{2}{3}$ indicates random, rapid, and isotropic rotational motion of dyes.^55,56^ However, evidence suggests that this assumption is not always accurate, and that the value of κ^2^ can vary heavily,^56^ particularly for structures where there are fluctuations in the conformation of the labeled biomolecule, such as a twisted DNA helix.^93^ Furthermore, the assumption that $\kappa^{2}=\frac{2}{3}$ implies that κ^2^ does not vary with the donor–acceptor distance.^56,94^ However, this is problematic as it has been shown that these variables are correlated,^94^ proving the assumption incorrect. It is also possible that incomplete orientational sampling occurs during individual bursts when photons are released.^56^ If the full rotational freedom during a burst is not explored, the FRET efficiency obtained will be biased by the specific orientations that were sampled within the short time. Therefore, κ^2^ will vary across different bursts, will not be held constant, and the assumption that $\kappa^{2}=\frac{2}{3}$ would become invalid. All things considered, using the commonly defined value of two-thirds for κ^2^ can lead to inaccurate calculations of *R*_0_ as a result of errors being introduced in the distance calculation, further resulting in incorrect interpretations of molecular conformations.

An aspect to consider when simulating a smFRET environment is the impact of solvent conditions on dye spatial confinement, which can influence dye motions and therefore FRET efficiency. Shoura et al. in 2014 ^82^ assessed the impact of solvent conditions on the spatial confinement of fluorophores and how the resulting spatial confinement affected the interpretation of experimental FRET data in Cre recombinase-DNA complexes. Their focus was primarily on exploring the effect of glycerol on dye-distance distributions and deviations from isotropic fluorophore motion. The study compared surface accessibility results from MD simulations to experimental work they have previously conducted.^95^

To accomplish this task, four different systems were simulated using MD simulations: (1) donor and acceptor fluorophores tethered to fixed points in space by six-carbon linkers, (2) a DNA duplex bearing the product of the Cre-recombination reaction from a previous experimental work where fluorophores are conjugated to C5 positions of adjacent thymine residues on opposing strands, (3) the Cre Holliday-junction intermediate complex with fluorophore labels at sites corresponding to the positions used in the experimental work, and (4) a fluorophore-labeled Cre-mediated synapse of DNA duplexes. To align closely with experimental work, they used ATTO 647N as the acceptor fluorophore, while the donor fluorophore was modeled using the known structure of ATTO 610. Six-carbon linker chains attached the fluorophores to the C5 positions of specific thymine residues.

The MD simulations were then performed in three stages. In the first stage, short simulations were run where the atomic positions of the backbones were constrained to their starting positions. In the second stage, the systems were allowed to relax with constraints applied only to the terminal base pairs of the DNA structures to prevent the fraying. In the third stage, where the data was collected, long simulation times were used. In this stage, data was gathered on the fluorophore positions, their orientations, and on time-dependent anisotropy for comparison with experimental results. The results revealed that dye-linker interactions can restrict fluorophore motion indicating that the freely rotating assumption may not hold true. They also found a significant difference in the dye-pair distance-distribution functions obtained for MD simulations carried out in water and in glycerol/water mixtures. In the glycerol containing simulations, the dyes had decreased mobility as a result of the increased viscosity of the solvent which altered the distributions and affected the obtained FRET efficiency values. By comparing MD-derived fluorophore spatial distributions to experimental FRET data, the study showed that static surface accessibility models fail to capture dye-solvent, dye–dye, and dye-macromolecule interactions. Incorporating solvent composition in MD-based modeling was suggested as a valuable approach for experimental design in future FRET studies.

Another significant challenge when simulating dyes can occur because fluorophores can potentially stack onto the labeled biomolecule.^83^ If this occurs, the conformation gets distorted which can lead to inaccurate FRET efficiencies. To address the stacking problem, in 2018 Grotz et al.^83^ employed TIP4P-D^96^ and TIP4P/2005(1.1)^97,98^ water models in their simulations which incorporate corrections for water–water and water-solute London dispersion interactions. The corrections increase the solvation of the dyes, preventing incorrect stacking and overall improves the accuracy of the dye’s behavior in the simulations.

The study used a maleimide-functionalized Alexa 594 that was covalently attached to a thiol linker attached at the 5′ end of the nucleic acid and a *N*-hydroxysuccinimide ester-functionalized Alexa 488 that was attached to an amino linker attached at the 3′ end of the nucleic acid. The performance of the dyes was evaluated in MD simulations with five water models (TIP3P,^99^ TIP4P-Ew,^100^ TIP4P/2005,^98^ TIP4P/2005(1.1),^97,98^ and TIP4P-D^96^) using thymine dinucleotides with single dyes attached. The trajectory analysis included the calculation of correlation functions, computation of orientation factors, FRET efficiencies, distance time traces, fluorescence anisotropy decays, and distributions. Additionally, a dye-mapping approach was developed to improve the accuracy of FRET efficiency predictions for single-stranded DNA and RNA under varying salt concentrations.

Their results showed that the dispersion correction in TIP4P-D^96^ and TIP4P/2005(1.1)^97,98^ water reduced extensive dye-base stacking in fluorophore-labeled nucleic acids, leading to a more accurate description of the fluorophore’s behavior. The dye-mapping approach also helped address deficiencies observed in explicit dye-labeling simulations, which often overestimated dye stacking and misrepresented the fluorophore’s dynamics. By improving the dye description, the MD simulations of single-stranded RNA were able to approximately reproduce the dimensions observed in smFRET experiments, and simulations of single-stranded DNA were able to capture the overall trend of salt sensitivity observed experimentally. Therefore, this study highlights that computational and experimental methods can be integrated together to advance understandings of biomolecules such as nucleic acids.

In a study conducted in 2015 by Best et al.,^85^ the focus was on parametrizing the Alexa Fluor 488 and Alexa Fluor 594 fluorophores which are commonly employed in single-molecule protein experiments in conjunction with the TIP4P/2005 ^98^ water model. The researchers derived a set of parameters for these fluorophores and validated them by comparing simulation results against experimental observables that are sensitive to dye-protein interactions. These included fluorescence correlation functions, fluorescence anisotropy decays, and FRET efficiencies. They found that scaling the protein–water interactions, an empirical method introduced to improve protein–protein association simulations, also enhanced the accuracy of dye-protein interactions. Additionally, they addressed a concern raised by molecular simulations regarding polyproline experiments, suggesting that approximately 20–50% of the chromophores may bind to the hydrophobic polyproline helix with average associated lifetimes of less than 10 ns.

For parametrization, the researchers employed standard AMBER atom types for the chromophore atoms, fixed the Lennard-Jones parameters, and introduced angle and torsion terms based on existing AMBER force fields. Atomic charges were determined using the restrained electrostatic potential (RESP) fitting method implemented in the ANTECHAMBER program, supported by quantum chemistry calculations. The aliphatic linker was also parametrized with atom types and charge assignments similar to related groups in AMBER force fields. To test the approach, simulations were performed using different combinations of protein force fields and water models, specifically TIP3P^101^ and TIP4P/2005.^98^ The protein force fields were based on the AMBER ff03 and AMBER ff99SB energy functions, with modifications to backbone torsion angles to ensure correct helix propensity for each water model.

Results showed that the TIP4P/2005 ^98^ water model, in combination with scaled Lennard-Jones interactions between the protein and solvent, provided the best agreement with experimental fluorescence anisotropy decays, which serve as sensitive probes of protein-dye dynamics. In contrast, the commonly used TIP3P model^101^ produced less accurate results. These findings suggest that refining Lennard-Jones interactions in force fields is necessary to improve the modeling of chromophore dynamics and dye-protein interactions. Here, the authors emphasized the importance of carefully optimizing force-field parameters for accurately interpreting smFRET experiments using MD simulations. The integration of MD simulations with experimental data provides insights into excluded volume effects and favorable chromophore–protein interactions, with the impact varying depending on the specific system under investigation. They concluded that the combination of smFRET and MD offers a deeper understanding of biological systems at the single-molecule level and highlighted the potential of machine learning approaches for future analysis of smFRET data.

Simulating an smFRET environment can also be challenging when incorporating heavy atoms of FRET dyes into molecular dynamics simulations. In 2018 Reinartz et al.^84^ addressed this issue by employing a coarse-grained (CG) approach (Figure 3) to simulating FRET-labeled proteins. The reduced computational cost of CG simulations allows for longer time scales to be accessible, improving the sampling of conformational ensembles to more accurately reflect the dynamic nature of the labeled biomolecule and the dyes. In turn, allowing for the analysis of complex dynamic ensembles with atomic detail with a minimal set of parameters. To validate their approach, they compared simulated FRET efficiency distributions against experimental histograms, demonstrating strong agreement. They also compared their model to the accessible volume approach and analytical polymer models for FRET descriptions, showing improved precision in predicting FRET efficiencies.

**Figure 3:**
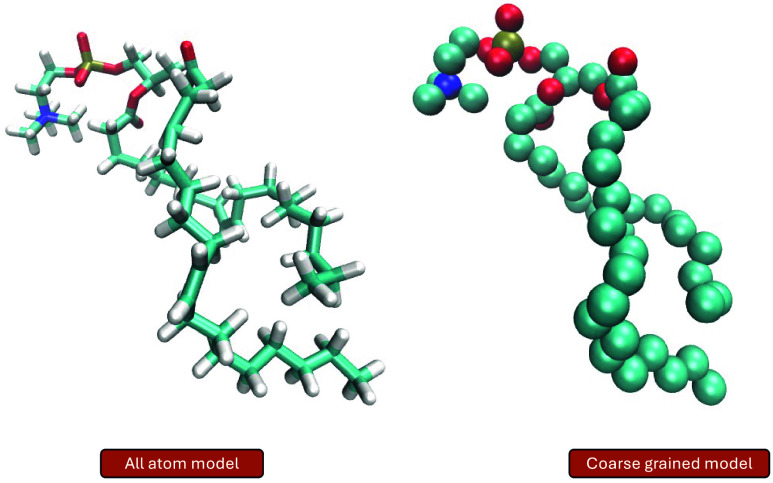
Schematic representation of using coarse-grained systems for MD simulations. In coarse-grained models, groups of atoms are represented as beads, reducing the system’s degrees of freedom. This simplification significantly accelerates simulations, enabling the study of larger systems and longer time scales while reducing computational cost in comparison to to all-atom MD simulations.

The study examined two dye pairs, Alexa Fluor 488 with C5-linker (AF488) and Alexa Fluor 594 with C5-linker (AF594), as well as Alexa Fluor 546 with C5-linker (AF546) and Alexa Fluor 647 with C2-linker (AF647). Quantum-chemical calculations were performed to provide the three-dimensional structures of the dyes, including linkers and maleimide groups for protein attachment. Their model treated dyes and proteins as distinct groups in the simulation, allowing them to be assigned separate temperature baths to accurately reproduce dye rotational dynamics. Three proteins were used as test systems in their study: chymotrypsin inhibitor 2, the tenth type III module of fibronectin, and the cold-shock protein from *Thermotoga maritima*. Dye attachment was simulated by replacing specific residues with cysteines and covalently linking the dye-maleimide structures, ensuring proper orientation while avoiding steric clashes. After determining appropriate dye temperatures and rotational correlation times by using time-dependent fluorescence anisotropy (eq 10) MD simulations were performed.

Donor and acceptor photons were generated for the simulated trajectory until a defined burst size and each of these bursts was used to calculate a single FRET efficiency value. By incorporating experimentally derived rotational correlation times of the dyes, their model captured fluorescence anisotropy decay and improved the accuracy of simulated FRET distributions. This method provided a direct link between structural ensembles and experimental FRET efficiency distributions, allowing researchers to systematically investigate parameters such as Förster radius, dye pairs, linker lengths, and labeling sites. The results showed that the simulated data aligned well with experimental data, providing valuable insights into biomolecular processes by complementing experimental FRET efficiency distributions with atomically resolved structural ensembles from simulations.

The studies discussed above demonstrate that integrating MD simulations with smFRET can be a powerful and reliable approach for studying the behavior of proteins and gaining insight into biological dynamics such as changes in protein conformation. However, to ensure reliability of the data interpretation, it is crucial to carefully consider how fluorophores are incorporated into MD simulations to accurately model their behavior. Factors such as solvent effects, fluorophore stacking, incorporation of heavy atoms, parametrization of the dyes, and more must be considered to ensure meaningful comparisons with experimental results.

### II.B Estimating FRET Efficiency from MD

Among several studies that have addressed the FRET efficiency estimation from MD simulations, here we focus on a few key studies that have provided valuable insights. In a study conducted by Best et al.^72^ in 2007 the researchers employed a combination of experimental techniques and MD simulations to interpret smFRET experiments with polyproline spacers. They first utilized nuclear magnetic resonance (NMR) spectroscopy to determine the fraction and position of cis prolines in the system. Single-molecule photon trajectories were then measured using pulsed, picosecond excitation of freely diffusing molecules, enabling accurate determination of FRET efficiencies from fluorescence decay curves. To interpret the experimental results, the researchers performed MD simulations that incorporated the dyes and their linkers. The simulations were used to analyze single-molecule lifetime and intensity data, integrating the population distributions of cis-proline residues obtained from previously conducted NMR experiments with simulated efficiency values. The obtained FRET efficiency histograms were compared to predictions using a theoretical model accounting for both photon statistics and background noise, providing validation of the experimental observations. In addition, measurements were conducted in trifluoroethanol to explore the effect of reduced cis proline content, expanding the study’s applicability across different solvent environments.

For the experimental data analysis, photon trajectories were recorded from dilute solutions of freely diffusing polyproline-20 molecules labeled with FRET donor and acceptor dyes. The trajectories were divided into time bins, where low-photon bins were excluded from further analysis. A correction was applied to account for differences in donor/acceptor quantum yield and detection efficiency by randomly deleting acceptor photons, as outlined in Nir et al.^102^ To quantify noise, the shot noise width was calculated using

5 $$\sigma_{s.n.}^{2}=\left\langle E\right\rangle \left(1-\left\langle E\right\rangle \right)\left\langle N^{-1}\right\rangle$$

where ⟨*E*⟩ is the mean FRET efficiency and *N* is the number of photons per time bin. Frequency distributions of consecutive donor and acceptor photons were calculated for each FRET efficiency interval. These distributions were normalized based on empirical photon counts per bin to correct for dye blinking effects. They found that if the order of detection of donor and acceptor photons is random, the probabilities of observing a sequence of consecutive acceptor photons (ν*_A_) or consecutive donor photons (ν_D_*) is simply given by (eq 6):

6 $$p\left(\nu_{A}\right)=E^{\nu_{A}}\;\mathrm{and}\;p\left(\nu_{D}\right)={\left(1-E\right)}^{\nu_{D}}$$

For the MD simulations, an implicit solvent model was employed to simulate the all-trans polyproline structure, given its repulsive interactions. However, to better capture the flexibility of the dye linkers, a five-proline fragment was attached to each dye in explicit solvent. This multiscale approach enabled efficient simulations while maintaining a realistic representation of dye conformational distributions. By integrating experimental data with MD simulations and theoretical analysis, Best et al.^72^ developed a robust framework for interpreting FRET efficiency histograms. Their results demonstrated that the presence of cis-proline residues significantly increased molecular flexibility, leading to higher FRET efficiencies than expected for a rigid polyproline helix. The consistency among NMR, smFRET lifetime and intensity measurements, and MD simulations results provided strong evidence that a quantitative understanding of smFRET experiments with polyproline spacers is achievable when accounting for noise and blinking effects.

In a study conducted by Hoefling et al. in 2011,^56^ the researchers developed an approach that avoided assuming the isotropic orientation factor to address the aforementioned challenges encountered under this assumption by incorporating dye orientation dynamics from MD simulations with experimental FRET efficiency distributions. To achieve this, they combined MD simulations of an Alexa 488 and 594 dye-labeled polyproline (15, 20, and 30-mer)^103^ in solution with Monte Carlo (MC) simulations of dye excitation, FRET, and fluorescence decay events.^56^ Their four-step approach began by employing all-atom MD simulations of multiple isomeric conformations, which were combined using a Boltzmann-weighted ensemble, to capture structural fluctuations. In the second step, the transition dipole moments of the donor and acceptor fluorophores were extracted from the simulations in order to calculate the orientation factor.The orientation factor is defined using the general definition in (eq 7). Figure 4 shows how the three angles that contribute to the orientation factor were defined.

7 $$\kappa^{2}\left(t\right)={\left[\cos\theta_{\mathrm{DA}}\left(t\right)-3\cos\theta_{\mathrm{DR}}\left(t\right)\cos\theta_{\mathrm{AR}}\left(t\right)\right]}^{2}$$

**Figure 4:**
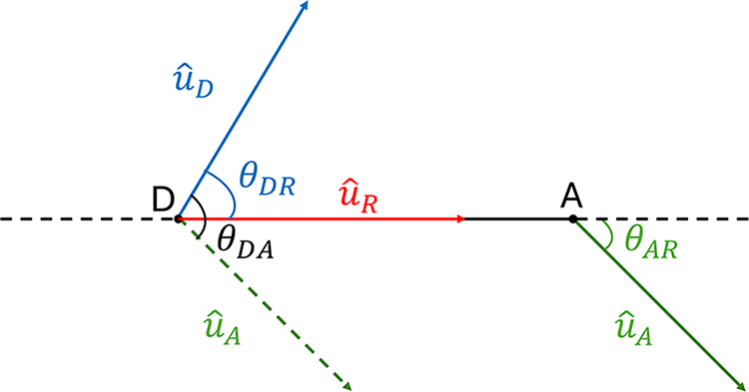
Schematic representation showing how the three angles that contribute to the orientation factor are defined. θ_DA_ is the angle between *û*_D_ and *û*_A_ (black) where *û*_D_ and *û*_A_ represent the unit vectors associated with the orientations of the donor and acceptor dyes, respectively. θ_DR_ is the angle between *û*_D_ and *û*_R_ (blue). θ_AR_ is the angle between *û*_A_ and *û*_R_ (green).

These orientations were then used to calculate instantaneous FRET rate coefficients, *k*_*T*_(*t*) (eq 8), which describe the probability (eq 9) that a FRET occurs at each moment of time, *t*. Since *k*_*T*_(*t*) depends on both the instantaneous electronic coupling between the dyes and their mutual orientations, their approach ensured that the FRET rate considered changes in dye alignment. Förster’s dipole approximation was applied to measure the electronic coupling between the donor and acceptor dyes. In this equation, the total donor decay rate is represented as the inverse of the donor fluorescence lifetime, 1/τ_D_. The donor lifetime, τ_D_, and the quantum yield, *Q*_D_, were used in the calculation of the instantaneous FRET rate coefficient (eq 8).

8 $$k_{T}\left(t\right)=\frac{1}{\tau_{D}}{\left(\frac{R_{0}}{r}\right)}^{6}$$

9 $$P_{T}\left(t\right)=\Delta t\cdot k_{T}\left(t\right)$$

In the third step, using the probability that a FRET occurs at each instant of time, *P*_*t*_(*t*), MC simulations were conducted to simulate and collect individual photon absorption and excitation, FRET, and emission events. For each photon absorption event, a random snapshot from the MD trajectories was chosen and the FRET probabilities were generated until a photon emission or decay event occurred. After averaging over many events, fluorescence intensities were calculated for the donor and the acceptor dyes leading to an average FRET efficiency value. In the final step, the emitted photons were gathered into bursts based on the experimental photon burst size distribution and FRET efficiency was calculated for each burst, allowing for comparison with experimental methods.

Employing their approach allowed the researchers to make direct comparisons of their constructed FRET histograms to experimental smFRET data. However, unlike methods that assume the isotropic dye orientation, their approach included κ^2^ averages calculated based on the MD of the system where changes in dye orientation were accounted for. By doing this, their approach took into account all possible correlations between dye motion and distance, thus allowing for precise mutual orientation distributions to be obtained and for the acquirement of improved geometrical information on the labeled biomolecule.

When simulating smFRET environments large molecular linkers are typically used to attach dyes to a biomolecule of interest allowing for large free rotation of those dyes. Therefore, the isotropic probability distribution is followed in these cases^80,104–106^ because the dyes are able to sample all possible orientations equally. However, additional problems can arise because smFRET is based on the ideal dipole approximation (IDA)^50^ in which the donor and acceptor fluorophores are treated as point dipoles that are small in comparison to the separation distance of the dyes on the labeled biomolecule. As a result of this, accurate structure prediction is possible only if the assumption of the IDA is valid.^80^ However, there are cases in which the assumption of the IDA will not be valid. For example, it is known that the IDA will fail when small donor–acceptor distances are present.^107^ In a work done by Spiegel et al. in 2016 ^80^ researchers investigated the failure of the IDA by analyzing the dynamics of a double-stranded RNA molecule tagged with Alexa Fluor 488 as the exciton donor and Cy5 as the acceptor at different positions. Their goal was to determine the extent to which deviations from the IDA affect FRET rates at short donor–acceptor distances.

To accomplish this goal, Cy5′s linker and the dye residues were placed on a fixed modified uracil base and a separate linker that carried the Alexa Fluor 488 dye was placed in varying positions. Center of mass distances between the dyes were calculated which were defined as the mass-weighted average positions of C9 and O10 in the xanthene ring of the Alexa Fluor 488 dye and C3 of the pentyl chain of the Cy5 dye used. The model dye structures were placed on top of snapshots of trajectories from all-atom MD simulations to analyze their spatial distributions and the dynamics of the dye orientations. To quantify deviations from the IDA, the Excitonic Coupling Matrix Element (ECME), which describes how strongly the transition dipoles of molecules influence each other, was calculated using the IDA and a monomer transition density approach. Deviations between the two methods were analyzed.

In all of their setups, the researchers identified certain positions where the dyes tended to be located which restricted free rotation in space and led to deviations from a perfectly isotropic distribution of the transition dipoles. They emphasized that the distance between the anchor points of the dyes did not directly imply how far the dyes can be located relative to each other due to flexibility of the linker. Therefore, to obtain a better estimate, the flexibility and the length of the linker must be considered when estimating dye separation. However, despite observing instances where the deviation from the IDA played an important role, it was found that the number of snapshots affected by a large deviation of the IDA represented only a small part of the total trajectories. As a result, they only had a minor statistical impact on the time-averaged ECME and excitation energy transfer rate. Therefore, the authors concluded that the IDA is acceptable for FRET experiments that have small donor–acceptor distances with the stipulation that dye attachment should not overly constrain motion.

Another crucial consideration to accurately describe dye behavior is to ensure correct descriptions of the rotational (Figure 5) and translational motion of the fluorophores used.^81^ In a study conducted by Deplazes et al. in 2011 ^81^ researchers sought to determine if MD simulations were able to correctly describe the motions of the fluorophores and to gain insight into the relationship between FRET efficiency and fluorophore separation distance. To accomplish this goal, the researchers used MD simulations to examine the rotational motion, translational motion, and FRET efficiency of independent fluorophores in solution. Since it is known that fluorophore motion should be unrestricted, the researchers were able to validate their computational findings against theory to show that MD simulations are able to accurately model fluorophore behavior.

**Figure 5:**
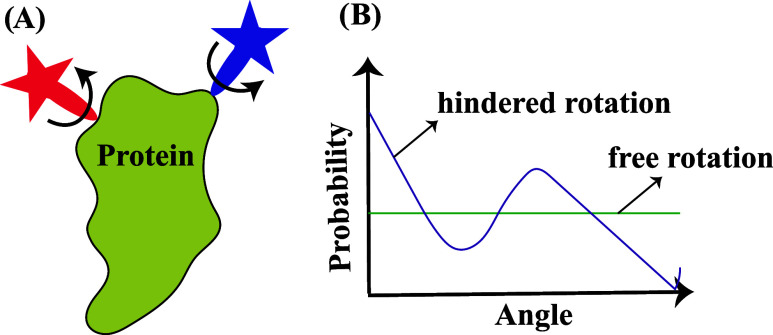
(A) Schematic representation showing rotational movement of the dyes on a labeled biomolecule where the dyes on a labeled protein (green) are depicted in blue and red. (B) Schematic representation of the probability distribution of dye angles based on free rotation (green) and hindered rotation (magenta).

The study modeled eight Alexa Fluor 488 (donor) and eight Alexa Fluor 568 (acceptor) molecules in a cubic box. The directions of the unit vectors for the transition dipole moments of the molecules were taken along the long axis of the outer rings in the head groups. Furthermore, the distance between the donor and acceptor dye was defined by the connection vector that joins the central oxygen in the headgroup of the dyes. To analyze the orientation factor, κ^2^, 64 donor–acceptor pairs were considered and followed eq 7. The FRET efficiency was determined using eq 2 by including the donors and acceptors in the central box and the acceptors in the surrounding boxes. In doing this, they ensured each donor was surrounded by a bulk solution of acceptors up to a range greater than 2*R*_0_. The study also calculated fluorescence anisotropy (*r*(*t*)) to compare MD predictions with experimental fluorescence data by using the following equation, where *P*_2_(*x*) = (3*x* ^2^ – 1)/2 is the second Legendre polynomial, μ̂(0) and μ̂(*t*) are unit vectors along the transition dipole moments (for either donor or acceptor) at the time of excitation (0) and at some time later (*t*) (eq 10):

10 $$r\left(t\right)=\frac{2}{5}\left\langle P_{2}\left(\mathrm{\mu ̂}\left(0\right)\cdot \mathrm{\mu ̂}\left(t\right)\right)\right\rangle$$

Here, the notation μ̂ is equivalent to the unit vectors *û* used in Figure 4, both representing the orientations of the transition dipole moments of the dyes. In eq 10, the time-average, shown via angle brackets, was calculated by assigning each frame in the simulation to *t* = 0 and calculating the mean of the resulting *r*(*t*) for a series of succeeding times 0 + *t*. Then, from this they determined rotational correlation time by fitting the final obtained *r*(*t*) to an exponential:

11 $$r\left(t\right)=r_{0}\;\exp\left(-\frac{t}{\tau_{\mathrm{rot}}}\right)$$

where *r*_0_ = 2/5 and τ_rot_ is the characteristic time scale.

Overall, they found that their computational model successfully reproduced the theoretical FRET efficiency vs donor–acceptor separation distance relationship for both the static and dynamic orientational averaging cases. Additionally, they also stressed that it is important to verify the MD simulation model to ensure that the force fields used to describe the individual dyes are well parametrized and that the setup and parameters of the simulation are reasonable, if not, fluorophore motion could be described incorrectly leading to inaccurate results for the FRET efficiency conclusions. This can be done by comparing the fluorescence anisotropy calculated from the simulation data to the experimental value. Here, the calculated fluorescence anisotropy values were consistent with experimental data, confirming the accuracy of the force fields used in the simulations. The results presented in this study imply that MD simulations can accurately describe the isotropic motion of fluorophores in solution, a key requirement for modeling FRET accurately and ensuring correct interpretation of the molecular conformation of the labeled biomolecule.

Together, these studies collectively contribute to the estimation of FRET efficiency from MD simulations, providing insights into integrating experimental techniques, deviations from ideal dipole approximations, and data analysis. They highlight the importance of integrating smFRET with MD to accurately model fluorophore dynamics, dye-linker flexibility, and more in biological systems at the single-molecule level. This interdisciplinary approach not only improves the reliability of FRET-based measurements but also lays a foundation for more precise and predictive models of biomolecular behavior.

## III Using MD to Gain Insight into Experimental Data

Conducting MD simulations and performing experimental techniques provides deeper insight into biomolecular systems and increases our understanding of dynamic processes. In particular, MD simulations have been used to complement experimental results, providing structural and dynamic information that is not easily accessible through experimental techniques alone. Similarly, experimental methods have been used as tools to guide and to validate MD simulations. The following section focuses on two key aspects of using MD to gain insight into experimental data: the validation of experimental smFRET data and the utilization of enhanced sampling methods.

### III.A MD and smFRET Use in Parallel

Using MD simulations to complement experimental data has become increasingly common because MD simulations can offer atomic level insights into the conformations of the biomolecule of interest and can be used as validation tools. To gain a better understanding of allosteric communication, in 2012 Wolf et al.^79^ examined allosteric regulation in the yeast heat shock protein 90 (Hsp90) following ATP hydrolysis. They employed fluorescence techniques, including smFRET, fluorescence correlation spectroscopy, and fluorescence lifetime measurements, in conjunction with MD simulations to probe conformational changes associated with ATP hydrolysis in Hsp90. The corrected FRET efficiency histograms revealed three predominant distance populations. To connect smFRET measurements and their MD simulations they calculated the expected distance distributions for fluorophore pairs directly from intraprotein dye-accessible volumes of the simulated structures. This allowed for a quantitative comparison between simulated conformational states and experimentally measured mean distances with their uncertainties. The combination of experimental measurements and MD simulations provided insights into the allosteric communication in Hsp90 and its connection to client protein binding and folding processes. The combined approach enabled a time-resolved investigation of the conformational changes underlying signaling and regulation, offering a more comprehensive view of Hsp90s conformational dynamics.

Expanding on the utility of integrating smFRET and MD simulations, Li et al.^108^ employed both methods to gain a deeper understanding of the early activation mechanism of the receptor tyrosine kinase MET in 2024. In their work, the researchers site-specifically labeled a protein called internalin B (InlB) which binds to MET. They were then able to measure donor–acceptor distances across the InlB-MET dimer interface upon InlB binding. These smFRET measurements revealed that InlB stabilizes MET in an extended conformation. Complementary to their experimental work, the researchers also ran MD simulations of the ectodomain of MET further providing evidence that MET exists in both a compact and an extended structure. Together, the integration of the methods allowed the researchers to determine that the stabilization of MET by InlB pushes MET to the extended conformation, a conformation critical for activation of the receptor and for the formation of MET dimers. By integrating smFRET and MD data, the researchers proposed a mechanistic model describing the early steps of MET activation at the membrane.

Another application employing both MD simulations and experimental methods was conducted by Borgia et al. in 2018 ^76^ where the researchers investigated the binding between prothymosin-α (ProTα) and histone (H1) proteins to investigate unstructured proteins that have physiological function. Overall, they were interested in determining the extent of structure formation that occurred when the proteins interacted together. In their study, experimental methods were employed to examine the formation of secondary and tertiary structures in ProTα and H1 individually and when they were bound to each other. Additionally, smFRET was utilized to quantify the strength of the interaction between the proteins. In their simulations a CG model was used for the proteins and separate CG simulations were run for the incorporation of the linkers and dyes which were represented by 5 beads. Overall, the integration of smFRET, other experimental methods, and molecular simulations provided a detailed structural representation of the H1-ProTα complex’s conformational ensemble. The findings presented in this study suggested that high-affinity complex formation between ProTα and H1 can occur in the absence of folded domains or structured regions of the proteins. Instead, they found that the interaction is driven by a rapid interconversion between multiple arrangements on a nanosecond time scale. Therefore, by using both experimental and computational methods, the researchers were able to provide a complete picture of how intrinsically disordered proteins are able to interact dynamically.

MD simulations and experimental techniques can also be integrated to investigate complex biological mechanisms such as gating in membrane transporters. In a study conducted by Levine et al. in 2019 ^77^ the researchers explored substrate-specific allosteric modulation of intracellular gating in sodium transporters by combining MD simulations, quantum mechanics/molecular mechanics (QM/MM) calculations, smFRET imaging, and *Na* ^+^ binding assays. Their study aimed to understand how substrate interactions with specific residues influence transporter dynamics, building upon hypotheses developed from their previous work on sodium transporters. All FRET efficiencies were calculated based on eq 1, where *E* was set to zero whenever the donor was in the nonemissive “dark” state where energy transfer could not occur, for example, due to temporary photoblinking or photobleaching. FRET traces were then fit to a three-state model using the segmental k-means algorithm.^109^ Their results indicated that the occupancy of specific conformational states was necessary for achieving functional states associated with rapid substrate transport. The integration of MD simulations and QM/MM calculations enabled a mechanistic understanding of how specific substrate interactions modulate transporter dynamics at an atomic level. On the other hand, smFRET imaging and *Na* ^+^ binding assays provided experimental validation of these findings, confirming the presence of substrate-dependent gating behaviors. The study exemplifies how computational and experimental methods can be synergistically applied to unravel complex mechanisms underlying transporter function.

Collectively, these studies illustrate the power of integrating MD simulations with smFRET experiments to uncover key aspects of allosteric interactions in solution, binding interactions, and protein conformational dynamics. The synergy between computational and experimental methods enables atomic-level characterization of dynamic processes that would be challenging to resolve using either approach alone. The combined strategy enhances the ability to probe biomolecular mechanisms, providing a deeper and more comprehensive understanding of protein structure and function.

### III.B Parallel Integration of smFRET with MC/MD Simulations

Beyond validation, several studies have leveraged smFRET data as a component of MC workflows. To gain a better understanding of the conformations of intrinsically disordered proteins, in 2012 Nath et al.^74^ conducted a study combining computational studies with experimental smFRET measurements using α-Synuclein (αS) and tau proteins in solution. The researchers developed and introduced an Enhanced Conformational Monte Carlo (ECMC) method to incorporate long-range pairwise distance constraints from smFRET into MC simulations. In this approach, the effective potential included repulsive Lennard-Jones interactions and harmonic restraints based on average distances and variances based on smFRET measurements.^110,111^ Additionally, the authors performed unconstrained MC simulations to broadly sample conformational space and generated ensembles of at least 400 distinct structures for each set of parameters.^74^

To validate their method, the researchers performed all-atom MD simulations of the αS protein in explicit water where the conditions closely mimicked the experimental smFRET conditions. Ten different initial configurations were randomly selected from the end states of ECMC simulations and were simulated for a total of 574 ns, with the first 10 ns of each trajectory omitted to allow for structural relaxation. The MD results confirmed that the ECMC approach accurately reproduced the global dimensions of αS observed in the smFRET experiments, demonstrating both the internal consistency of the smFRET data and the effectiveness of ECMC as a novel tool for investigating the conformational behavior of disordered proteins.

Similarly, in a study conducted by Melo et al. in 2016 ^75^ researchers employed smFRET and computational simulations to investigate the structural characteristics of the intrinsically disordered tau protein, however, in this study tau was bound to soluble tubulin heterodimers for the purpose of determining domain-specific conformational changes upon binding. For the smFRET experiments, they introduced specific cysteine residues at desired locations and labeled them with maleimide fluorophores (Alexa Fluor 488 and Alexa Fluor 594).

The MC simulations were designed to incorporate the experimental smFRET constraints, estimating the mean and standard deviation of the inter-residue distance distribution that would yield a specific effective transfer efficiency (ET_eff_) for an unconstrained chain. The ET_eff_ values were then converted into distance using the following equations where $\langle r^{2}\rangle^{1/2}$ is the root-mean-square distance (eqs 12–14):

12 $$ET_{\mathrm{eff}}=\int_{0}^{\infty }E\left(r\right)P\left(r\right)dr$$

13 $$\mathrm{where},P\left(r\right)=4\pi r^{2}{\left(\frac{3}{2\pi \left\langle r^{2}\right\rangle }\right)}^{\left(3/2\right)}\exp\left(-\frac{3r^{2}}{2\left\langle r^{2}\right\rangle }\right)$$

14 $$\mathrm{and},E\left(r\right)=\frac{1}{1+{\left(\frac{r}{R_{0}}\right)}^{6}}$$

Their results showed that tubulin binding resulted in localized expansion within individual repeats of tau’s microtubule-binding region, but that the overall dimensions of the protein remained largely unchanged. This supports a model in which tau retains significant intrinsic disorder, acting as a flexible scaffold with multiple binding sites for tubulin or microtubules. The combined use of smFRET experiments and MC simulations was essential in revealing these nuanced, domain-specific structural changes. The smFRET data provided precise, experimentally measured distance constraints between specific residues, while the MC simulations integrated these constraints to explore the full spectrum of tau’s conformational states. Ultimately, the integrated approach used was essential for uncovering domain-specific structural variations that might have been overlooked using either method alone.

Experimental smFRET and MD simulations can also be used synergistically to investigate the structural dynamics of isoforms. In a study completed by Meng et al. in 2018 ^78^ researchers utilized smFRET and MD simulations to evaluate the dynamics of Aβ40 and Aβ42 peptides, two key isoforms involved in amyloid-related diseases. They site-specifically labeled the N- and C-termini of the peptides with Alexa 488 and Alexa 647 by integrating an unnatural amino acid called 4-acetylphenylalanine.^112,113^ smFRET measurements were performed in solution and on a glass surface enabling a comparative assessment of behavior under different conditions, for both peptides, and the results were compared. Additionally, they conducted both conventional and temperature replica-exchange MD simulations on the isoforms of interest in explicit solvent. The results of their work from the experimental methods and the simulations showed good agreement in that most of the conformations were disordered and had only local structure to them.^78^ This study demonstrated the use of a combined smFRET and MD simulation approach to characterize conformational heterogeneity in intrinsically disordered peptides and highlighted subtle structural differences between the isoforms.

The examples mentioned above illustrate how smFRET and MC simulations can be used to explore the conformational landscapes of intrinsically disordered proteins and peptides. The synergy described not only reveals structural features of the molecules of interest, but enhances the variability of biologically relevant structures as a result of MC approaches being able to rapidly transverse conformational landscapes.

### III.C Use of Enhanced Sampling Methods

Enhanced sampling methods (Figure 6) play a crucial role in exploring the conformational space of biomolecules and capturing rare events that are often beyond the reach of traditional MD simulations. These techniques typically manipulate simulations by introducing a biasing potential on collective variables that describe the system’s behavior. In a study conducted by Merchant et al. in 2007 ^73^ the researchers investigated the unfolded states of proteins using a combination of smFRET spectroscopy and molecular simulations. They focused on protein L and protein CspTm, aiming to obtain quantitative insights into their size, dynamics, and folding mechanisms. To label the recombinant protein L, Cys residues were introduced at the N- and C- termini and were reacted with maleimide derivatives of Alexa Fluor 488 and Alexa Fluor 594. The labeled proteins were purified and smFRET efficiencies for both proteins were measured. Fluorescence bursts were identified by dividing the data into 1 ms bins and merging adjacent bins containing at least nine photons into individual burst events.^73^

**Figure 6:**
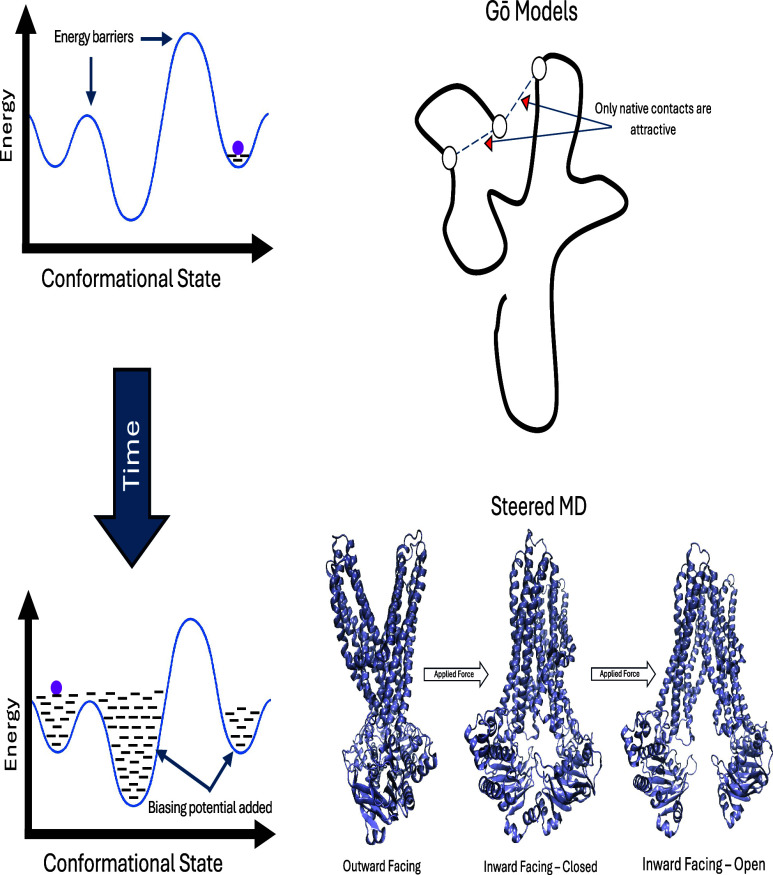
Schematic overview of three enhanced sampling techniques commonly used in biomolecular simulations. Left: Schematic representation of metadynamics. A bias potential is constructed by periodically adding repulsive Gaussian hills along selected collective variables, allowing the system to escape local minima and explore rare conformational events. Top right: Go̅-like model, which biases the energy landscape by favoring native contacts. Bottom right: Steered MD, in which an external force is applied along a reaction coordinate to induce transition from outward-facing to inward-facing state.

In the molecular simulations performed in the study, a Go̅-like energy function was employed to model protein folding. Additionally, Langevin simulations used a simplified model for the polypeptide structure in which each amino acid residue was represented as a spherical bead. All-atom MD simulations of the unfolded proteins in a cell of urea and water were also run. To assess statistical uncertainties, block error analysis was used to estimate the impact of sampling errors. Their findings showed strong agreement between the mean FRET efficiency of the folded protein obtained from the Langevin simulations and the experimentally measured values. Further analysis of the simulations and experimental donor fluorescence decays for the unfolded proteins revealed that, within the donor’s lifetime, the dyes experienced complete orientation averaging, but the polypeptide backbone remained essentially static. With this in mind, the researchers highlighted that unfolded proteins exhibit dynamics on various time scales and emphasized the importance of investigating dynamics occurring after 1 μs.

Similarly, Girodat et al.^114^ employed a Go̅-like structure-based simulation approach in 2020 to provide structural and molecular insights into dynamic biomolecular events at an atomic level. Their goal was to quantitatively compare in vitro biochemical and spectroscopic data with in silico MD simulations, allowing for a direct structural interpretation of conformational processes associated with ligand binding and unbinding. The study established a pipeline for recreating smFRET data in silico, using the LIV-BP system as a case study to bridge experimental observations with computational models. The Go̅-like structure-based simulation approach allowed for the definition of multiple FRET states as native basins in the simulation, facilitating interconversion between these states.^115^ This allowed for a time-scale comparison between MD simulations and smFRET data. The researchers used all-atom structure-based simulations aligned with in vitro experiments and explicit solvent simulations on shorter time scales to investigate key variables and determinants of smFRET data, including the quantitative descriptions of the *R*_0_ and fluorophore orientation factor parameters that provide insights into the FRET-distance relationship.

The MD simulations explicitly modeled LD555 and LD655 fluorophores at the same positions as in the experimental setup. The conformational changes of LIV-BPSS (a mutated version of LIV-BP) in smFRET experiments with varying camera frame rates were examined. For the MD simulations of LIV-BPSS, site-specifically labeled proteins were used in dual-basin all-atom structure-based simulations to capture conformational changes between open and closed conformations. Explicit solvent simulations were conducted for both apo and Leu-bound states to explore the dynamics of LIV-BPSS near the open and closed basin minima.

To quantitatively compare experimental and simulated smFRET data, the researchers measured the distance between the centers of mass of the explicit LD555 and LD655 chromophores attached to positions 67 and 181 of the protein, respectively, in the MD simulations. Using the estimated duration of simulated time steps and the *R*_0_ value, they generated simulated FRET efficiency traces. The mean FRET efficiencies and standard deviations of experimentally measured FRET states were determined by compiling individual smFRET traces into population FRET histograms, which were then fitted to Gaussian distributions. The study demonstrated that the simulated dynamics of LIV-BPSS closely matched the experimental observations through smFRET, validating their pipeline for integrating MD simulations with experimental smFRET data to study ligand-induced protein conformational changes. In their approach, they were able to validate their model and gain mechanistic insight into the transitions between the conformational states, this would not have been possible if both experimental and computational approaches were not employed.

In a study conducted in our laboratory in 2021 by Baucom et al.^116^ researchers employed a unique combination of experimental and computational biophysical techniques to investigate how the intrinsically disordered C-terminal region of Albino3 (Alb3) contributes to its role in protein targeting and membrane insertion. The study utilized smFRET, circular dichroism, site-directed mutagenesis, trypsin digestion assays, and all-atom MD simulations with enhanced sampling techniques. A detailed model of Alb3-Cterm in a fully extended conformation at the atomic level was created and 16 different conformations of Alb3-Cterm were generated using a MC algorithm. These conformations were used for 16 well-tempered metadynamics simulations^117^ to explore conformational flexibility for 320 ns. The α-variable was used as the collective variable in all metadynamics simulations to quantify the α-helical propensity of a protein or peptide of length N using (eq 15):

15 $$\alpha =\frac{1}{2}\left(\frac{1}{N-2}\Sigma_{n=1}^{N-2}f\left(\theta_{n}\right)+\frac{1}{N-4}\Sigma_{n=1}^{N-4}g\left(d_{n}\right)\right)$$

In eq 15, θ_*n*_ is the angle formed by *C*_α_^(*n*)^– *C*_α_^(*n*+1)^ – *C*_α_^(*n*+2)^ and *d*_*n*_ is the distance between *O* ^*n*^ and *N* ^(*n*+4)^. The *f*(θ) and *g*(*d*) are score functions quantifying the likelihood of θ and *d* being associated with an α-helix (eq 16) where θ_0_ and *δθ* are 88 and 15°, respectively, and *d*_0_ is 3.3 Å.

16 $$f\left(\theta \right)=\frac{1-{\left(\frac{\theta -\theta_{0}}{\delta \theta }\right)}^{2}}{1-{\left(\frac{\theta -\theta_{0}}{\delta \theta }\right)}^{4}}\;\mathrm{and}\;g\left(d\right)=\frac{1-{\left(\frac{d}{d_{0}}\right)}^{6}}{1-{\left(\frac{d}{d_{0}}\right)}^{8}}$$

The helical propensity of individual amino acids based on the MD trajectories was calculated and the 16 trajectories were combined to generate per-residue helical propensity profiles. Simulations using Alexa488 and Alexa594 dyes were performed in explicit water for 100 ns, producing 136,000 simulated conformations of Alb3-Cterm with dyes attached. Distance distributions derived from MD were then compared with smFRET experimental data, revealing excellent agreement between the computational and experimental interfluorophore distances.

This study employed a novel combination of smFRET and all-atom MD simulations that take advantage of enhanced sampling techniques to identify local transient secondary structure in the intrinsically disordered C-terminal region of the Alb3 insertase. The findings suggest that the Alb3-Cterm, despite its disorder, contains structural elements that may play a role in recognizing and binding the transit complex. These results underscore the power of combining experimental and computational approaches to characterize dynamic, disordered protein regions with functional significance.

In a study conducted in our laboratory in 2022 by Benton et al.^118^ the structural characteristics of the complete cpSRP43 protein were investigated in terms of stability and flexibility. The researchers employed a combination of computational methods, including equilibrium and nonequilibrium all-atom MD simulations at the microsecond level, as well as experimental techniques. Equilibrium MD simulations were run to determine the stability of cpSRP43 compared to the cpSRP43/cpSRP54 complex and nonequilibrium steered MD simulations were run based on previously published small-angle X-ray scattering (SAXS) data.^119,120^ They steered their docked model toward the SAXS conformation using distance vectors as the collective variables. The ending conformation was used to generate the next model, and the process was repeated until four models were obtained each representing a different conformation of the SAXS model. These models were equilibrated and simulated. Then, smFRET data was used to validate the conformations. The researchers found that their comparative MD simulation data on cpSRP43 and the cpSRP43/cpSRP54 complex were in qualitative agreement with previously published experimental smFRET data, providing further evidence for the validity of their computational models. This study successfully identified a stable conformation of monomeric cpSRP43, revealing intrinsic flexibility and dynamic behavior essential for its function. By integrating microsecond-level MD simulations, SAXS-guided modeling, and experimental validation, the researchers provided new structural insights into cpSRP43 that would have been challenging to uncover using a single technique alone.

The integration of MD simulations with smFRET and enhanced sampling techniques has proven to be a powerful strategy for investigating protein dynamics, conformational transitions, and structural flexibility. Traditional MD simulations are often limited in sampling slow conformational changes, but enhanced sampling techniques help overcome these limitations by efficiently exploring rare events and conformational states. When combined with smFRET, these computational approaches provide a direct way to quantitatively interpret experimental distance distributions and validate conformational ensembles observed in experiments.

### III.D MD Based MSM with smFRET Experiments

MD simulations have the ability to explore submillisecond time scales and can therefore provide crucial insights into biomolecular interactions that complement the static information obtained from experimental structures. By capturing dynamic motions, MD simulations offer a deeper understanding of specific regions of interest in proteins, shedding light on their functional mechanisms. Integrating smFRET measurements into MD simulations allows for the interpretation and enhancement of simulation results, providing a more physiologically relevant description of biomolecular behavior. Durham et al.^121^ highlighted the potential of combining smFRET with MD simulations for gaining insights into structural dynamics in their 2020 work. By capturing dynamic motions, MD simulations provide valuable information about specific regions of interest in proteins that cannot be inferred from static structures alone. The authors emphasized the role of smFRET measurements in constraining MD simulations to enhance their physiological relevance. While current studies focus on time scales of seconds to milliseconds, the future of smFRET research lies in investigating submillisecond dynamics to address unresolved questions.

As previously discussed, using a CG model can extend the simulation times achievable by MD simulations, however, problems such as degeneracy of conformations need to be addressed. In a study conducted by Matsunaga et al. in 2015 ^45^ the researchers integrated smFRET photon-counting data into CG MD simulations using a data assimilation process^122^ to overcome problems such as data being degraded by linker fluctuations. They developed a framework based on particle filtering, which included running numerous replicated MD simulations, to accurately identify conformational transitions. The framework formulates a likelihood function that connects photon-counting data with the structural characteristics of a target molecule. Specifically, they used end-to-end distances and donor–acceptor distances from the trajectory and simulated photon emissions as Poisson processes to bin the photon emission data. The joint probability of detecting *N*_*A*_ acceptor and *N*_*D*_ donor photons in a time bin of length *T* is defined in eq 17:

17 $$P\left(N_{A},N_{D}|\mathrm{conformation}\;\mathrm{in}\;\mathrm{time}\;\mathrm{bin}\;T\right)=\left[\frac{{\left(\mathrm{nT}\right)}^{N_{A}+N_{D}}}{\left(N_{A}+N_{D}\right)!}e^{-\mathrm{nT}}\right]\left[\frac{\left(N_{A}+N_{D}\right)!}{N_{A}!N_{D}!}{\mathrm{E̅}}^{N_{A}}(1-\mathrm{\epsilon ̅}{)}^{N_{D}}\right]$$

where *n* is the total expected number of photon-counting rate from the acceptor and donor per unit time and *E̅* is a time average of FRET efficiency, *E*, over the time bin (eq 18):

18 $$\mathrm{E̅}=\frac{1}{T}\int_{0}^{T}E\left(t\right)dt=\frac{1}{T}\int_{0}^{T}{\left(1+{\left(\frac{r\left(t\right)}{R_{0}}\right)}^{6}\right)}^{-1}dt$$

*R*_0_ generally could be a function of κ^2^, which could be time-dependent as well; however, in this study, the authors have eventually made the rapid, isotropic sampling assumption for the dye rotation to simplify the calculations.

In each cycle of the particle filter, MD simulations were performed for each particle to obtain the predictive density. Then, the filtering process was applied to the predictive density using the likelihood function. Overall, 40 cycles of the particle filter were conducted to obtain a dynamically corrected ensemble based on the emulated smFRET photon-counting data MD simulation. The initial distribution, *P*(*x*_0_), was generated through MD simulations incorporating a distance restraint between the donor and acceptor dyes to ensure realistic starting conformations. To evaluate the performance of the data assimilation using smFRET photon-counting data, the researchers used data from a CG MD simulation of a dye-labeled polyproline-20 (*Gly*(*Pro*)_20_ *Cys*).

The results of the study indicated that their method successfully recovered hidden conformational transition events from smFRET data, even in the presence of linker fluctuations, which typically obscures these transitions. Furthermore, the approximations obtained were robust against variations in model parameters, provided the prediction errors remained sufficiently small. However, the authors noted that a challenge that was encountered was the disparity between experimental smFRET time scales and simulation time scales. One potential solution to bridge this gap is to increase the number of photons in each time bin in smFRET experiments, thereby enhancing the statistical accuracy and compatibility with CG simulation time scales.

To address the time-scale gap between single-molecule experiments and MD simulations, Matsunaga et al.,^86^ employed a novel approach based on MSMs^123^ for analyzing single-molecule time-series data in 2018 (Figure 7). This method fine-tuned the transition-probability matrix of a MSM using high-resolution single-molecule data. Specifically, they used a machine-learning approach to estimate *T*(τ) between hidden Markov states from low-dimensional time-series data.^124^ To accomplish this, a two-step procedure was proposed to combine machine learning and simulation data with single-molecule experiments. In the first step, an initial MSM was constructed using simulation data combined with supervised learning. For this process, extensive MC MD simulations were performed on a dye-labeled (Alexa 488, Alexa 594, and linkers) formin-binding protein (FBP) WW domain where the dye labeling mimicked experiment, totaling approximately 400 μs of simulation time. The simulations utilized a two-dimensional space defined by the native contact, and the expected FRET efficiency for MSM construction. Spatial clustering was applied and MC searches were conducted during the simulation process to ensure proper dye labeling without steric clashes.

**Figure 7:**
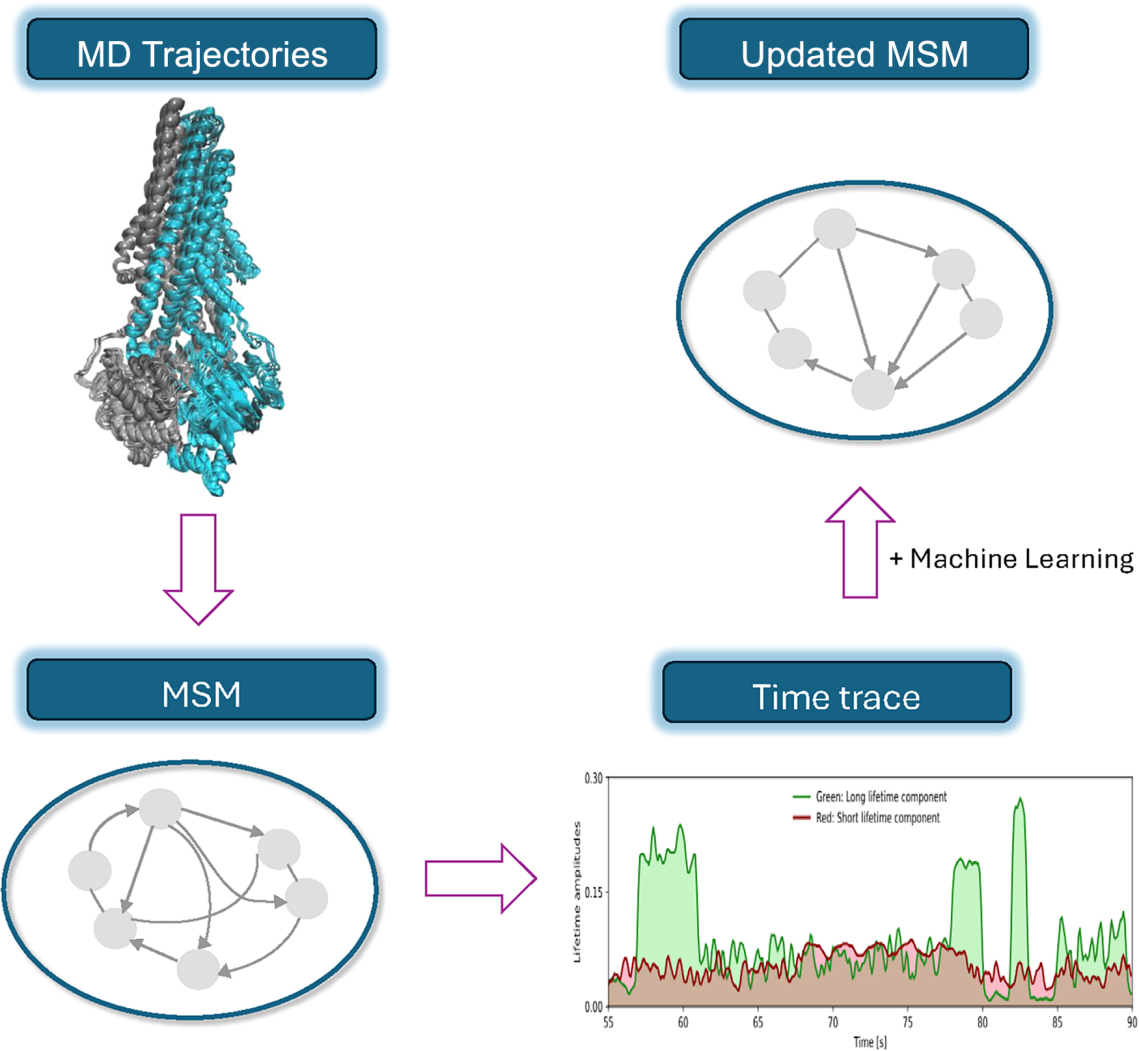
A schematic showing the integrative approach of combining Markov State Modeling (MSM), smFRET data, and machine learning algorithms to refine and validate MD trajectories.

In the second step, the initial MSM used in the first step was refined using hidden Markov modeling by incorporating single-molecule measurement time-series data. High time-resolution smFRET measurements were used in the unsupervised learning step to provide data on WW domain folding and unfolding dynamics. Photon trajectories for the FBP WW domain were measured using donor and acceptor fluorophores attached to the terminal residues of the protein. The photon color (green for donor or red for acceptor) and the absolute arrival times were recorded with a resolution of approximately 0.5 ns. Each photon trajectory was divided into folded and unfolded segments based on the photon interval with the highest transition probability. The final data set consisted of 527 smFRET photon sequences, each representing a single folding or unfolding event. Stochastic simulations of the refined MSM evaluated its dynamic properties, and the mean squared error between simulated and experimental FRET histograms ensured the model’s robustness against overfitting.

They found that the data-assimilated MSM effectively reproduced the experimental smFRET data and produced a transition-state ensemble consistent with an independent mutational experiment. Incorporating temporal information from experimental time-series data into simulation-based models provided a comprehensive and experimentally validated understanding of the folding mechanism of the FBP WW domain, demonstrating that this integrated semisupervised learning and MSM approach can effectively bridge experimental and simulation time scales.

In their subsequent work in 2018, Matsunaga et al.^87^ compared the performances of two possible methods for refining the MSMs using CG simulations of a dye-labeled polyproline-20.^58,72,125^ They performed two separate MD simulations: one based on a correct force field and one based on an intentionally inaccurate force field, whose torsional angle parameters were scaled. Then, two MSMs were constructed, one from each of the simulations. The MSM built using the simulation data with the inaccurate force field was refined using either time-series trajectory data or ensemble-averaged data derived from the MSM generated with the correct force field. This approach aimed to mitigate biases in MD simulation results due to inaccurate force-field parameters.

The refinement process approach involved two steps: (1) construction of an initial MSM based on MD simulation trajectories and (2) refinement of the MSM parameters using experimental measurements and machine learning methods. For refinement, simulated smFRET-like time-series trajectory data and ensemble-averaged data were generated using the correct MSM, and empirical distributions of the distances between donor and acceptor FRET dyes were calculated for each MSM state. The inaccurate MSM parameters were then optimized through hidden Markov modeling by maximizing a likelihood function based on these simulated data sets. They then analyzed their data to determine accuracy.

They found that machine learning refinement utilizing time-series data was successful in determining equilibrium populations of conformational states and that their transition probabilities were more accurate compared to refinement using ensemble-averaged data alone. Overall, their method provided more reliable estimations of concealed conformational states, however, accurately estimating transition probabilities between minor states could still pose some challenges.

In conclusion, the integration of MD simulations, smFRET experimental data, and MSMs with machine learning techniques holds great potential for developing a deeper understanding the structural dynamics of biomolecules. The integration significantly enhances the physiological relevance and accuracy of computational models by effectively incorporating experimental single-molecule data.

## IV Summary

Integrating MD simulations with smFRET experiments is gaining traction and can offer novel insights into the structural dynamics of biomolecules. The data from smFRET experiments can be paired with MD simulations to provide molecular level information that cannot be observed using experimental techniques alone. For characterizing highly dynamic systems, simplified or CG methods have been utilized for a range of subjects, including intrinsically disordered proteins, nucleic acids, and extensive chromatin arrays. This approach helps to more efficiently traverse the conformational landscape of complex systems. The studies discussed in this review demonstrate the effectiveness of MD simulations in simulating and aiding smFRET experiments by considering factors such as dye orientations, photon absorption, FRET, and emission events. By incorporating realistic dye properties, solvent conditions, and dye-macromolecule interactions, MD simulations provide valuable insights into spatial confinement, dye-distance distributions, and FRET efficiencies. The importance of accurate force field parametrization and interpretation of MD simulations is emphasized to ensure reliable estimation of FRET efficiencies. These simulations are validated using various experimental techniques, such as NMR spectroscopy and fluorescence decay curves to obtain a quantitative understanding.

Furthermore, the integration of smFRET data into MD simulations allows for the refinement and interpretation of computational models, enhancing their physiological relevance. Incorporating long-range pairwise distance constraints from smFRET measurements enables the generation of ensembles of structures that better represent the conformational space explored by proteins in solution. MD simulations have been successfully applied to investigate the conformations and dynamics of various proteins, leading to valuable insights into their functional mechanisms. Additionally, enhanced sampling methods, such as MC simulations and metadynamics, in combination with smFRET experiments have facilitated the exploration of rare events such as unfolded protein states, folding dynamics, and complex biomolecular interactions. MSMs have also been employed to analyze single-molecule time-series data and incorporate experimental measurements into MD simulations. By integrating machine learning techniques with MSMs, researchers have refined models and extracted three-dimensional structural information on latent states. This integrated approach bridges the gap between experimental and simulation time scales, providing a more comprehensive understanding of biomolecular dynamics.

Despite these advances, several challenges remain in directly comparing MD simulations with smFRET experiments. Quantitatively linking simulated interdye distances and orientation dynamics to experimentally measured FRET efficiencies remains nontrivial, since the accuracy of such comparisons depends on the parametrization of the dyes, the treatment of linker flexibility, and adequate conformational sampling.^80,84^ However, MD simulations could be used to guide the selection of optimal labeling sites for smFRET experiments by identifying residues that are sensitive to functionally important structural transitions. Additionally, emerging artificial intelligence-driven models could enhance smFRET–MD integration by accelerating conformational sampling and identifying predictive structural features, expanding the predictive power of these hybrid methodologies.

Further progress in this field will benefit from bridging the gap between experiment and simulation which can be facilitated by continuous improvements regarding imaging platforms and fluorophore developments, enabling smFRET studies to be carried out in the microsecond time domain. Additionally, reduced computational burdens at these time scales will allow for simulations to run longer. As computational resources extend simulated time scales, the divide between experimental and simulation approaches will narrow. These synergistic efforts deepen our understanding of complex biological systems and the structural dynamics underlying conformational transitions observed in smFRET experiments. Ongoing focus on this frontier is expected to improve the development of integrating MD simulations with smFRET, expanding the predictive capabilities of the techniques across diverse areas of research.

## Data Availability

No new data is reported in this review article.
